# Infection control measures in times of antimicrobial resistance: a matter of solidarity

**DOI:** 10.1007/s40592-020-00119-9

**Published:** 2020-11-07

**Authors:** Babette Rump, Aura Timen, Marlies Hulscher, Marcel Verweij

**Affiliations:** 1 National Coordination Centre for Communicable Disease Control, RIVM-Centre for Communicable Diseases, Postbus 1, 3720 BA Bilthoven, The Netherlands; 2 National Coordination Centre for Communicable Disease Control, RIVM-Centre for Communicable Diseases, Bilthoven, The Netherlands; 3 Scientific Center for Quality of Healthcare (IQ healthcare), Radboud Institute for Health Sciences, Radboud university medical center, Nijmegen, The Netherlands; 4 Section Communication, Philosophy and Technology, Wageningen University, Wageningen, The Netherlands; 5 Athena institute for Research on Innovation and Communication in Health and Life Sciences, VU University Amsterdam, Amsterdam, The Netherlands

**Keywords:** Amr, Ethics, Solidarity, Isolation, Infection control measures, Justification

## Abstract

Control measures directed at carriers of multidrug-resistant organisms are traditionally approached as a trade-off between public interests on the one hand and individual autonomy on the other. We propose to reframe the ethical issue and consider control measures directed at carriers an issue of solidarity. Rather than asking “whether it is justified to impose strict measures”, we propose asking “how to best care for a person’s carriership and well-being in ways that do not imply an unacceptable risk for others?”. A solidarity approach could include elevating baseline levels of precaution measures and accepting certain risks in cases where there is exceptionally much at stake. A generous national compensation policy that also covers for costs related to dedicated care is essential in a solidarity approach. An additional benefit of reframing the questions is that it helps to better acknowledge that being subjected to control measures is a highly personal matter.

## Introduction

The rise of antimicrobial resistance is a pressing public health problem. Without effective antibiotics, infections that today are considered mild and easy to treat will pose a serious threat in the future. Antimicrobial resistance is not a faraway doom scenario: mortality due to resistant pathogens is estimated as high as 23,000 deaths a year in the United States and 25,000 in the European Union [1, 2].

Antimicrobial resistance is a complex phenomenon rooted in an evolutionary reality and accelerated by human behavior on many levels. Therefore policies to control antimicrobial resistance include many strategies, ranging from new drug development to judicious use of the still available stock of effective antibiotics, and preventing the spread of already resistant microorganisms into healthcare related settings—where they can reach those patients who are most vulnerable to infection.

The latter requires that people who are found to carry a resistant microorganism are subjected to infection control measures upon hospitalization. These control measures fit a long tradition of outbreak management. Measures like isolation and quarantine have proven their worth during many outbreaks throughout the last century and, more recently, during the SARS and Ebola outbreaks. Notwithstanding their effectiveness, they put at stake important ethical values like autonomy, freedom and well-being. Consequently, control measures directed at carriers are considered an ethical issue [3].

The issue is traditionally depicted as a trade-off between public interests on the one hand and individual autonomy on the other [4, 5]. The ‘public’ side of this trade-off involves promoting and protecting the health of the public, maximizing health benefits and preventing harm. The ‘individual’ side is about respecting freedom and autonomy and protecting other interests of the individual. Justification of control measures essentially takes the public health measures as a starting point and can be brought back to the question ‘is it justified to impose strict control measures on people for the sake of preventing disease in others?’.

The control measures directed at carriers of resistant microorganisms rely on the same tradition. Patients in high-risk settings such as hospitals and nursing homes who are found to carry a resistant microorganism, are placed in isolation and healthcare workers wear protective clothing (gowns, gloves, masks) when caring for them [1, 2, 6, 7]. These measures aim to regulate introduction and further transmission of resistant microorganisms into healthcare; they are necessary because hospitalized patients with serious illness and severe comorbidity are at the highest risk of infection due to resistance [8-10]. As can be expected, these measures also have burdensome implications for the carriers: they are reported to cause stigma, compromise quality of care and impact well-being [11-15].

The issue of control measures thus seems a conflict in which the health of vulnerable patients must be balanced against quality of life of (suspected) carriers. Prioritizing the protection of the vulnerable hospitalized patients then seems the obvious thing to do: mortality and morbidity rates leave no doubt about the impact of resistant microorganisms for these vulnerable persons while the precautions will not be life-threatening for carriers, only impact some aspects of their well-being, and are—in the end—only temporary. Reducing the issue of control measures directed at carriers to a binary trade-off is, however, unsatisfying in the complex case of antimicrobial resistance.

We propose to reframe the ethical issue and consider control measures directed at carriers an issue of solidarity. Rather than asking “is it justified to impose strict control measures to prevent AMR transmission from carriers to vulnerable others”, one could ask “how can we best care for this person’s carriership and well-being in ways that do not imply unacceptable risk (of transmission) for other patients?”.

## Shared interests and shared responsibilities

Because the rise of AMR is a complex and multi-faceted problem that is driven by human behavior on many levels in society [16]. Resistance is found in humans, animals and the environment (including soil and wastewater); all of these reservoirs contribute to the epidemiology of resistance [17]. In fact, all use of antibiotics contributes to emergence of resistance, although a major factor is underuse, overuse and misuse. Within healthcare, inappropriate antibiotic use and failure to apply effective infection control measures are key drivers. In agriculture, misuse and overuse are important drivers [16-18]. Ultimately, all human beings contribute to the problem of emerging resistance.

Likewise, keeping resistance rates low is something that concerns all of us. Medicine has become highly dependent on antibiotics. Antibiotics are used therapeutically (to treat patients with infections) but their applicability goes beyond that. Prophylactic use (e.g. during surgical intervention) reduces complications caused by bacterial infection. Moreover, modern medical achievements like chemotherapy, organ transplantation or neonatal care largely rely on the availability of antibiotics [19]. The traditional binary approach does not do justice to AMR being a shared responsibility, hence creating the need to also share the burden more equally.

The issue of people being colonized by a resistant microorganism and in this way being a threat to vulnerable hospitalized patients, can thus not be seen in isolation from our shared responsibility for causing microorganisms to become resistant and our shared interest in effective antibiotics.

## The known and the unknown

Another good reason to broaden our perspective is that the current situation seems somewhat arbitrary. Resistance is increasingly present in the community and it is infeasible to identify and treat all people that carry a resistant microorganism [1, 2]. Moreover, resistance primarily threatens a specific subgroup of vulnerable patients in hospitalized settings, therefore screening almost exclusively targets people who need to be hospitalized and are thought to be at high risk of colonization [8-10]. This at minimum includes people who suffer from infection caused by a resistant microorganism. In many countries (like the Netherlands and Scandinavian countries) this screening also includes patient that share a household with a known carrier, were previously admitted in a foreign hospital, or work with livestock that is associated with high prevalence of resistance [1, 2, 6, 7]. At the same time, due to overall prevalence of resistance, at any given moment in time a proportion of the hospitalized patients is also colonized with a resistant microorganism but not screened and thus unrevealed. If known carriers face severe restrictions, and unknown carriers do not, this may not only be burdensome and stigmatizing to them – such restrictions could also be considered rather unfair.

The situation shows large similarities with the early days of the HIV epidemic in 20th century. HIV started out as a life threatening incurable disease, and precaution measures within healthcare were essential to prevent HIV from transmitting to other patients and hospital staff. Initially HIV screening policies almost exclusively addressed high-risk patients, with specific backgrounds and profiles, and this had burdensome, stigmatizing implications for those groups. Over time, the dominant perspective shifted and today all patients (regardless background and profile) are considered potential carriers. Universal precaution measures are strict and this includes workplace safety regulations to prevent *all* bloodborne infections. As a result extra measures specifically addressing patients infected with HIV or high-risk groups for infection are hardly necessary in regular health care settings.

## Personal needs and individual preferences

Reframing the ethical issue has an extra added benefit. Putting the focus on optimal care for carriers (while taking into account risks for others) helps to bring to the front what truly is at stake here and what we thus should be concerned about protecting. Traditionally, justification of control measures in infectious disease control relies on evaluation of impact of the measures in terms of individual autonomy. This autonomy is usually understood as some form of liberty, emphasizing on self-determinations and self-governance, stating that ‘patients are only truly autonomous if they are able to act, intentionally, with understanding and without controlling influences that determine their action’ [5]. This kind of autonomy is hardly at stake here. In our research we found that some hospitalized carriers perceive isolation as an interference with their freedom, but most are bound to bed anyway and may enjoy the privacy of isolation or are indifferent about it [11]. Carriers seem more concerned about quality of care while in isolation, the unprofessional and stigmatizing behavior of healthcare staff, or they perceive isolation as unpleasant and boring [12]. Even after hospital discharge, when people are not subjected to control measures anymore (and autonomy as such is not at stake), knowledge of being a carrier still has a large impact on daily life. People feel hesitant to hold and hug their (grand)children because of fear of passing on the resistant microorganism and they perceive stigma and social exclusion [11]. The measures directed at carriers of resistant microorganisms thus put at stake more and different things than autonomy as such [3]. At the same time, the burdens people do experience, are a highly personal and contextual matter, so their personal perspectives are certainly relevant [3, 11]. The traditional approach does not do justice to AMR being this shared responsibility and thus the need to share the burden more equally. Asking how to best care for this carrier in ways that do not imply unacceptable risk helps to share the burden and enables us to better acknowledge the personal needs and preferences of each individual carrier.

## A solidarity approach

In sum, the emergence of antimicrobial resistance and the problems it creates for health care practice cannot be seen in isolation from our collective responsibility for the causes and the spread of resistance. The issue should not be reduced to a matter of balancing risks for those who are vulnerable to infections against the autonomy and wellbeing of carriers. Rather than having a policy that is primarily burdensome for carriers, or one that is with more risk for vulnerable patients, we plea for antimicrobial resistance policies that involve burdens that can and should be shared by society at large, as a matter of solidarity.

Our shared interest in effective antibiotics, our collective responsibility for antibiotic resistance, and the fact that identified carriers are only a subset of a larger group that can spread resistant pathogens, are strong reasons to be cautious with control measures that put a high burden on identified carriers. The issue calls for a paradigm shift and a change in clinical practice.

## Share the burden

We could consider shaping our policies in such way that the burden of preventive measures are more fairly spread among all of us. In fact objective 3 (elevating hygiene and infection prevention measures) and 4 (promoting stewardship and optimal use) of the *WHO’s global action plan on antimicrobial resistance* are good examples of a solidarity approach [20].

Standard precaution measures usually consist of hand hygiene (including limitations to the use of jewelry), routine environmental cleaning, appropriate waste management and the use of personal protective clothing when indicated [21]. From a solidarity perspective we could choose to consider all patients potential carriers and raise the baseline levels of precaution measures by adding, for instance, gown, gloves and mask, single rooms and private bathrooms, and mandatory social distancing for all patients. We could even ask all visitors to adhere to such strict measures. When all patients are considered to potentially be infectious, specific measures addressing only the carrier would hardly be necessary. Drawing conclusions on exact effectiveness of these measures is difficult because they are always installed in combination with other measures and because of conflicting data in published literature, but overall they are considered effective in controlling transmission of multidrug resistant organisms in healthcare settings [6, 21-23]. Creating such high levels of standard precaution measures though, is a costly matter and hospitals with single rooms and private bathrooms are a scarcity. Thus, reasonable as it may sound from a solidarity perspective, it might be unfeasible in many settings. What remains ethically relevant though, is that considering all patients as carriers will lead to a better commitment to infection prevention measures and take away some of the stigma experienced by carriers [24]. Moreover, any elevation of overall infection control measures will make some extra measures in exceptional cases more acceptable.

To take away some of the arbitrariness of the current situation we could also consider to subjecting all patients to screening upon entering the hospital. This will be associated with higher costs, partly because of costs of screening and mostly because more carriers will be exposed and thus subjected to the measures. Clearly, such a screening has limitations in terms of scope and impact. Moreover, new and unexpected resistant microorganisms will always emerge. Furthermore, this may eventually exacerbate instead of diminish stigma. Still, measures are not merely justified because they do justice to ‘effectiveness in preventing risk of harm’ but they are justified because they do justice to our shared responsibility. One could argue that, as a principal objective, treating equal people equally and subjecting equal people to equal measures is ethically more defendable.

## Compensate for the loss

This idea of treating equal people equally may be ethically appealing in terms of shared interests and shared responsibility; in terms of mitigating introduction and spread of resistant microorganisms into healthcare, the impact of people, notably potential carriers, simply is not equal. From a solidarity perspective, it can therefore instead be argued to collectively carry the burden by compensating carriers for their social and financial costs. This first, and foremost, should include compensation for financial loss. The measures are known to come with high costs for carriers due to direct costs of screening, eradication treatment and equipment (such as gowns and mask), indirect costs because of postponement of medical procedures, time consuming trips to the infection control clinic, and loss of income, notably for healthcare personnel [3, 11]. Such expenses can reasonably be covered in a collective national compensation policy.

## Centralize the individual experience

Yet, there is far more at stake here for carriers that should be considered for compensation, notably quality of care and wellbeing [3, 11]. We could shape precautions towards carriers a genuine attitude of hostmanship and care, offering the best possible health care, and ensuring full comfort and convenience, thus making their stay a first class experience. In the context of the reported delays in care and the reported unprofessional, uncaring and stigmatizing behavior of healthcare personnel, this seems the least we can do. Such dedicated care could be shaped by ways of a case manager or case ambassador who remains available throughout the care process and sees to it that the carrier does not lose out. When this ambassador also remains available after discharge, the confusion, stigma and loss in wellbeing experienced in daily life is also tackled. A generous national compensation policy that also warrants for costs related to dedicated care thus is essential in a solidarity approach.

It is true that concerns about wellbeing can also be addressed in the traditional approach. Specifically when one pays careful attention to things such as proportionality and always choosing the least intrusive means, the negative impact on wellbeing can be tackled. However in the traditional way of thinking the wellbeing of the carrier is only a final concern, whereas we argue that, because of this shared responsibility, the wellbeing of the carrier should be a leading argument. By asking ‘how to best care for all stakeholders’ wellbeing becomes a primary concern.

## Accept certain risks

Moreover, committing to a solidarity approach creates the much needed room to accept certain risks in those exceptional cases where there is disproportionally much at stake for the carrier. It is not hard to imagine what it means in terms of child development when medical daycare facilities separate carriers from the other children, or what it means for disoriented elderly inhabitants of nursing homes to be condemned to their rooms because of the measures [3]. We know of cases in which a medical student discontinued the education because of chronic MRSA colonization, and of young healthy children being subjected to very intensive MRSA eradication treatments in order for their parents to keep their job in healthcare [3, 25]

Please note that this does not mean that we should abandon all liberty restrictive measures, indeed the question ‘whether it is proportionate to impose strict measures’ remains relevant in both approaches. However in our way of formulating the central normative question, one takes the fact that we live in times of AMR and that this inherently comes with a certain amount of risk as a starting point. From thereof, in the end, one may indeed still reach the more specific question whether a specific liberty restricting measure is proportionate in a specific situation; and one may even conclude that it is. The traditional way of formulating the central normative question however, is much more related to zero-risk tolerance. Only in the end one may conclude that ‘in this specific situation this specific liberty restricting measure is not proportionate’ and that one therefore needs to accept a certain amount of risk.

A solidarity approach thus commits us to a language of inclusiveness, shared interest and shared responsibility. This enables us, if all other options fall short, to accept certain risks and distribute the burdens and benefits more equally over all people involved. In the end there is a new reality: multidrug-resistant organisms are without doubt one of the most pressing health threats of our time, but they are here to stay.

## Conclusion

Mitigating the spread of antimicrobial resistance is our shared interest and shared responsibility and thus an issue of solidarity. A solidarity approach should centralize wellbeing and the individual experience. From a solidarity perspective we should consider elevating the baseline level of precaution measures and accepting certain risk in cases where there is exceptionally much at stake for carriers. A generous national compensation policy that also warrants for costs related to dedicated care is essential in a solidarity approach. After all, we are not at war with resistant microorganisms, and certainly not with the people hosting them, instead we need to offer fair, responsible and sustainable care in times of antimicrobial resistance.
